# Associations between the Type of Tobacco Products and Suicidal Behaviors: A Nationwide Population-Based Study among Korean Adolescents

**DOI:** 10.3390/ijerph18020367

**Published:** 2021-01-06

**Authors:** Youn Huh, Hong-Jun Cho

**Affiliations:** Department of Family Medicine, Asan Medical Center, University of Ulsan College of Medicine, Seoul 05505, Korea; higjdus1@naver.com

**Keywords:** electronic cigarettes, heated tobacco products, suicidal thought, suicidal plan, suicidal attempt, adolescents

## Abstract

The relationships between multiple tobacco products, such as heated tobacco products (HTPs), electronic cigarettes (ECs), and combustible cigarettes (CCs), and suicide-related behaviors among adolescents have not been extensively researched. This study examined the associations between the type of tobacco products used and suicidal thoughts, plans, and attempts among Korean adolescents. Data from the 2019 Korea Youth Risk Behavior Web-Based Survey were analyzed, and participants included 57,069 individuals aged 13–18 years. A multivariable logistic regression analysis was performed. Of the total participants, 13.0%, 4.0%, and 2.9% reported suicidal thoughts, suicidal plans, and suicidal attempts, respectively. After adjusting for confounding variables, all tobacco product users showed a greater likelihood of having suicidal behavior. However, compared with never users, dual users of CCs and HTPs were not significantly associated with having suicidal thoughts and attempts. Among tobacco product users, dual users of ECs and HTPs and triple users of CCs, ECs, and HTPs showed a greater likelihood of having suicidal behavior. Considering the prevalence of suicide and the increasing trend of using multiple tobacco products among Korean adolescents, tobacco control policies should monitor the effects of different products.

## 1. Introduction

Suicide is a leading cause of death among adolescents and young adults worldwide and in South Korea (hereafter, “Korea”) [1,2,3]. The incidence of suicide in 2018 was 5.8 per 100,000 persons, which has not decreased since 2007 [4]. Additionally, the suicide rates have remained unchanged despite the clinical, scientific, and political efforts to improve the methods of predicting and preventing suicides [5]. Several epidemiological studies have reported an association between smoking and suicidal behaviors in adolescents [6]. A recent study demonstrated the association of electronic cigarettes (ECs) with depressive mood, mental illnesses, and suicidal behaviors [7,8]. One study found that about 34% of people with suicidal thoughts have plans for suicide, about 72% of people who plan for suicide actually attempt suicide, and a quarter of the people who have suicidal thoughts attempt suicide impulsively without having a premeditated plan [9]. Therefore, suicidal ideation and suicidal behaviors are important mental health issues among adolescents [10]. Thus, it is essential to assess the possible risk factors and approach individuals with suicidal thoughts appropriately. With this, as part of the efforts to predict and prevent adolescent suicide, there is an evident need for the active intervention of risk factors in adolescents [11,12].

The usage rate of combustible cigarettes (CCs) among American adolescents has decreased from nearly 40% in 1995 to about 10% in 2018 [13]. Meanwhile, the usage rate of CCs among adolescents in Korea has decreased by half since 2012 [14]. However, ECs and heated tobacco products (HTPs) have since then been introduced into the market [15,16,17]. In Korea, where ECs were introduced into the market in 2007 and extensively regulated, its use by adolescents has remained stable at less than 3% [14]. The prevalence of HTP use among Korean adolescents was 2.9% (4.4% for men and 1.2% for women) one year after the introduction of HTPs in the Korean market in 2017, and increased to 4.7% (7% for men and 1.9% for women) in 2019 [18]. The prevalence of HTP use among Korean adolescents is worrisome, as it is escalating quicker than EC use [19]. Additionally, the prevalence of the combined use of tobacco products has increased in adolescents using CCs because of the availability of alternatives [18,20]. CC alternatives are marketed as less harmful nicotine delivery devices, and the number of users of multiple tobacco products is increasing, although the physical and psychological safety of these devices has not been established yet.

Only a few studies demonstrate the association between the HTP and the combined use of these products and their association with suicide-related behaviors among teenagers. Therefore, we examined suicide-related behaviors among Korean adolescents according to the type of tobacco products used and the combined use of these products.

## 2. Materials and Methods

### 2.1. Data Source and Study Participants

Data were drawn from the results of the Korea Centers for Disease Control and Prevention’s 2019 Korea Youth Risk Behavior Web-based Survey (KYRBWS), known as KYRBWS-ⅩⅤ—an anonymous self-report online survey. Since 2005, the KYRBWS-ⅩⅤ has been conducted in June each year. It is implemented to calculate the statistics of health behaviors of Korean adolescents and develop a data source for the planning and evaluation of youth health policies and health promotion projects. The survey includes 105 questions on 15 aspects such as smoking status, alcohol consumption, and physical activity [14]. The KYRBWS provides a sample that is representative of the entire Korean middle and high school student population. The sample selection was performed in three stages: (1) stratification, (2) sample allocation, and (3) stratified cluster sampling. Through this process, 57,303 participants aged 13–18 years from 800 schools (400 middle and 400 high schools) were initially included in the study. However, individuals with any missing variables (*n* = 234) were subsequently excluded. The final analysis included the data of 57,069 adolescents (29,694 males and 27,375 females). All participants were assigned a unique identification number and answered an online questionnaire.

### 2.2. Ethical Considerations

Our study followed the principles of the Declaration of Helsinki. The KYRBWS is a statistical survey approved by the Korean government (Approval Number 117058). We requested permission from the Korea Centers for Disease Control and Prevention to use the KYRBWS results for our research. The requirement of informed consent was waived for the KYRBWS, as it uses anonymous and de-identified information.

### 2.3. Assessment of the Use of Tobacco Type

This study considered three tobacco products: CCs, ECs, and HTPs. Students were considered to have a history of CC use if they answered “Yes” to the question, “Have you ever smoked a cigarette in your lifetime, even one puff?” On the other hand, students were considered to have a history of EC or HTP use if they answered “Yes” to the question, “Have you ever used an EC or HTP (e.g., IQOS, glo, or lil) in your life?” A respondent was classified as a current CC, EC, or HTP user when the question, “How many days have you used at least one CC (or EC or HTP) in the last 30 days?” was answered as more than once a month. If a participant reported using a CC (or EC or HTP), but not within the past 30 days, he or she was classified as a “former CC user” (or a “former EC user” or a “former HTP user”). A participant who had never used a CC, EC, or HTP was classified as a “never user”. Thereafter, the participants were classified into nine groups: (1) never users; (2) former users of any of the three tobacco products; (3) only-CC users; (4) only-EC users; (5) only-HTP users; (6) dual users of CC and EC; (7) dual users of CC and HTP; (8) dual users of EC and HTP; and (9) triple users of CC, EC, and HTP.

### 2.4. Assessment of Suicidal Behaviors

In the survey’s mental health section, participants responded to the following questions: “Have you seriously considered suicide during the past 12 months?”; “Have you planned to commit suicide during the past 12 months?”; “Have you attempted suicide during the past 12 months?” Individuals who answered these questions with a “Yes” were classified as (1) considered to commit suicide, (2) planned to commit suicide, and (3) attempted to commit suicide, respectively.

### 2.5. Covariates

Self-perceived socioeconomic status was divided into five groups based on the economic status of the participants’ homes (high, high-middle, middle, low-middle, and low). Risky alcohol drinking was defined as the consumption of an average of ≥5 and ≥3 glasses of soju (Korean liquor) per occasion by males and females during the past 30 days, respectively [21]. Exercise levels were determined by the question, “How many days in a week do you engage in 60 min of physical activity that increases your heart rate more than usual or causes you to become breathless?” The participants could choose either “0 to 4 days” or “5 to 7 days”. Substance abuse was determined by the following question: “Have you ever taken substances such as butane gas, glue, and so on, habitually or deliberately?” The adolescents who responded “Yes” were determined to be engaging in substance abuse. Self-reported scholastic performance was divided into “Lower” and “Upper”. Subjective health status was categorized into “Bad” and “Good”. Presence of stress was determined by the following question: “How much stress do you usually feel?” If the participants responded, “I feel much”, “I feel a lot”, and “I feel a little”, they were classified as “Stressed”. If they answered, “I don’t feel much” or “I don’t feel at all”, they were classified as “Not Stressed”. Lastly, violence was assessed by the following question: “In the last 12 months, have you ever been treated in a hospital due to reasons caused by violence in relation to/associated with friends, seniors, or adults?” The adolescents who responded with “More than once” were classified as having experienced “Violent events”.

### 2.6. Statistical Analysis

Raw data from the 2019 KYRBWS were combined according to the KYRBWS raw data analysis guidelines. Moreover, based on a complex sample design, we conducted statistical analyses by assigning a dispersed stratification estimation, stratification variables, and weighted sample values. A chi-squared test was used to compare baseline characteristics of study participants and to evaluate proportions of suicidal behaviors according to the type of tobacco use. Thereafter, we determined the association between the type of tobacco use and suicidal behaviors by conducting a multivariable logistic regression analysis and calculating the odds ratio (OR) and 95% confidence intervals (CIs) by comparing adolescents who had never smoked and only-CC users. The multivariable logistic regression analysis was adjusted for sex, grade, subjective health status, income, risky alcohol drinking, lack of exercise, scholastic performance, and stress. Statistical significance was set at a *p*-value of less than 0.05 (*p* < 0.05). All analyses were performed using SPSS 24.0 (IBM Corp., Armonk, NY, USA).

## 3. Results

### 3.1. Baseline Characteristics

Table 1 shows the participants’ basic characteristics. About 7.7% of adolescents engaged in risky alcohol drinking. Of all participants, 80.9% were stressed and 2.3% had experienced violent events. Additionally, 86.3% (81.0% males and 92.1% females) of participants were never users. Among the adolescents using cigarettes, the proportion who were former users of tobacco products was the highest at 8.9% and 3.9% in boys and girls, respectively. The percentage of adolescents using only CCs was the next highest with 4.3% and 2.3% in boys and girls, respectively, followed by the triple users (2.4% in males and 0.7% in females) and dual users of CCs and ECs (1.4% in males and 0.5% in females).

### 3.2. Prevalence of Suicidal Behaviors According to the Type of Tobacco Use

Table 2 shows the basic characteristics and the prevalence of suicidal behaviors by the type of tobacco use. Of the total participants, 13.0%, 4.0%, and 2.9% reported having suicidal thoughts, plans, and attempts, respectively. The prevalence of suicidal thoughts, plans, and attempts in girls (17.1%, 4.9%, and 4.0%, respectively) was higher than that among boys (9.3%, 3.0%, and 1.8%, respectively) (*p* < 0.001). Statistically significant differences were found in the prevalence of suicide-related behaviors according to income, scholastic performance, alcohol consumption, substance abuse, self-rated health status, stress, and violent events (*p* < 0.001).

The frequency of suicidal thoughts, plans, and attempts for never users of tobacco products was 11.8%, 3.3%, and 2.2%, respectively. Suicide-related behaviors were more frequent among triple users than never users. Former users (18.3%, 6.2%, and 4.4% for suicidal thoughts, plans, and attempts, respectively) and current users of any tobacco product showed higher prevalence of suicidal thoughts, plans, and attempts than never users. Among them, the prevalence was highest in dual users of ECs and HTPs (32.3%, 20.1%, and 17.6% for suicidal thoughts, plans, and attempts, respectively). Consequently, statistically significant differences were found in the prevalence of suicide-related behaviors according to the type of tobacco use (*p* < 0.001).

### 3.3. Multivariate Analysis between the Type of Tobacco Use and Suicide-Related Behaviors

Table 3 presents the multivariable logistic regression analysis results of the association between the type of tobacco use and suicide-related behaviors. Compared with never users, dual users of EC and HTP had a significantly higher likelihood of having suicide-related behaviors (OR, 95% CI: 3.91, 2.12–7.24 for suicidal thoughts; 6.21, 3.14–12.31 for suicidal plans; 8.76, 4.11–18.70 for suicidal attempts). Compared with never users, the likelihood of suicidal thoughts and plans was the highest among triple users of CCs, ECs, and HTPs (OR, 95% CI: 3.08, 2.53–3.75 for suicidal thoughts and 3.75, 2.86–4.92 for suicidal plans), while that of suicidal attempts was the highest among only HTP users (OR, 95% CI: 7.13, 3.17–16.06 for suicidal attempts). In comparison with never users, the dual users of CCs and HTPs were not significantly associated with having suicidal thoughts and plans.

Compared with the adolescents using only CCs, suicidal thoughts, plans, and attempts were significantly higher in triple users of CCs, ECs, and HTPs (OR, 95% CI: 1.73, 1.41–2.12; CI: 2.23, 1.66–3.00; and CI: 2.59, 1.84–3.65, respectively), while suicidal plans and attempts were significantly higher for dual users of ECs and HTPs (OR, 95% CI: 2.89, 1.47–5.66 and CI: 3.00, 1.45–6.23, respectively).

## 4. Discussion

Our cross-sectional analysis of a nationally representative survey of Korean adolescents revealed that adolescents using any tobacco product (i.e., CCs, ECs, and HTPs) had a high prevalence of suicide-related behaviors. Former and current users of any type of tobacco products, including CCs, ECs, and HTPs, were more likely to report suicidal behaviors as compared with never users, except for dual users of CCs and HTPs, who were not significantly associated with having suicidal thoughts and plans. Moreover, compared with the only-CC users, triple users of CCs, ECs, and HTPs had significantly increased suicidal thoughts, plans, and attempts.

Several studies have demonstrated the association between smoking and an increased risk of suicidal behavior and suicide mortality. One study found that daily cigarette smoking predicted subsequent suicidal thoughts and non-fatal suicide attempts, after adjusting for confounding variables, such as prior depression and substance use disorders [22]. A meta-analysis determined the potential association between tobacco use and the risk of suicide mortality and revealed that the risk was 1.8 times greater for current cigarette smokers compared with never smokers [23]. Additionally, a recent study found that the use of ECs among adolescents increases suicidal thoughts, plans, and attempts by 1.6, 2.4, and 2.4 times, respectively [8].

The mechanism underlying the relationship between nicotine and suicide remains unclear; however, the following reasons can be considered. First, nicotine exposure disrupts the cerebral dopamine pathway, amplifies stress sensitivity, and distorts the coping mechanisms that buffer against depressive symptoms [24], particularly during adolescence when the developing brain is more vulnerable to nicotine-induced neurobiological insults [25]. Furthermore, depressive moods are also associated with suicide [9,26]. Additionally, nicotine is a strong activator of the hypothalamic–pituitary–adrenal (HPA) axis [27], and this HPA axis is thought to play a role in suicide risk [28]. This is supplemented by a relative hyperactivity of the HPA axis during adolescence, which may possibly increase suicide attempts in the individual [29]. Another explanation is that the mechanism associated with smoking and suicide is also involved in tobacco-related diseases such as asthma, allergic rhinitis, and atopic dermatitis [30,31,32]. Therefore, it should be noted that physical illness is a risk factor of suicide [33]. The trait of seeking novel products, such as ECs and HTPs, may contribute to the increased tendency toward suicidal behaviors in adolescence even after controlling for any psychopathologies such as depression [34,35]. Moreover, novelty-seeking behavior was specific for a suicidal group of bipolar patients [36]. High levels of novelty-seeking behavior in adolescence are associated with both externalizing disorders and suicide risk [37]. A study has even found that novelty-seeking is associated with dopamine activity and is implicated in the pathogenesis of psychotic disorders [38]. One study found that sensation seeking contributes to the risk of suicide attempts over and above the contribution of drug abuse such as tobacco [39].

The risk of suicidal behaviors was significantly increased in dual users of ECs and HTPs and triple users of CCs, ECs, and HTPs in adolescents, compared with only-CC users. Other studies showed that dependence on nicotine was higher among users of multiple tobacco products than among single tobacco product users [40]. In addition, a higher nicotine dependence and dose–response relationship was found between smoking and suicide, and the risk of suicide was increased by 24% for each increment of 10 cigarettes smoked per day [23]. It was supposed that dual users of ECs and HTPs and triple users of CCs, ECs, and HTPs in adolescents have an increased risk of suicidal behaviors compared with only-CC users because of nicotine dependence [40] and high levels of novelty-seeking behavior. There was no significant increase in suicidal thoughts and plans among dual users of CCs and HTPs, which is thought to be due to the small number of adolescents using them.

Public policy against cigarettes can reduce smoking among adolescents. One study using data on 717,543 adolescents in the 1999–2013 in United States showed that smoke-free legislation was an effective strategy to reduce smoking among all adolescents [41]. A recent study has shown that cigarette tax increases and enactment of 100% smoke-free restaurant were associated with reductions in cigarette use in adolescents [42].

Although this study provides reliable basic data on the type of tobacco use and suicidal behaviors in a representative sample of Korean adolescents, it has certain limitations. First, owing to the nature of cross-sectional studies, this study cannot explain causal relationships between tobacco use and the various suicide-related relevant variables among adolescents. Furthermore, we evaluated only the presence of suicidal behaviors and did not consider their frequency. Second, the data might be susceptible to some bias because the participants’ smoking status was self-reported. Additionally, we could not consider nicotine dependence because it was not included in the survey. Third, we could not perform a detailed survey to assess suicidal thoughts and plans as we relied on previously developed questions. Furthermore, though we considered some factors that influence suicidal behaviors, we could not account for all the confounding variables such as psychiatric disorders. In KYRBWS, the use of ECs and HTPs was investigated using examples of each product, but adolescents who confuse them with CCs were not completely considered. Despite these shortcomings, the present study included a large and representative sample of Korean adolescents.

## 5. Conclusions

The present study demonstrates the association of smoking with the risk of suicidal behaviors in adolescents. Adolescent users of ECs and HTPs have a higher risk of suicidal behavior compared with CC users. In addition, adolescent users of multiple tobacco products, especially the dual use of ECs and HTPs and the triple use of CCs, ECs, and HTPs, have the highest risk of engaging in suicidal behaviors. Therefore, it is necessary to raise awareness about this issue in schools and communities and adopt tobacco policies to control all types of tobacco products. In addition, further studies should determine the suicide-related risks longitudinally to establish a causal relationship.

## Figures and Tables

**Table 1:** Characteristics of study participants.

		Total	Boys	Girls	*p*
*N*		57,069	29,694	27,375	
Grade	Middle school	47.9 (0.8)	47.8 (1.5)	48.1 (1.6)	0.910
	High school	52.1 (0.8)	52.2 (1.5)	51.9 (1.6)	
Self-reported scholastic performance	Good–middle	68.2 (0.3)	68.5 (0.4)	67.9 (0.4)	0.207
	Bad	31.8 (0.3)	31.5 (0.4)	32.1 (0.4)	
Risky alcohol drinking	Yes	7.7 (0.2)	8.0 (0.3)	7.4 (0.3)	0.099
	No	92.3 (0.2)	92.0 (0.3)	92.6 (0.3)	
Substance abuse	Yes	1.0 (0.0)	1.0 (0.1)	0.9 (0.1)	0.203
	No	99.0 (0.0)	99.0 (0.1)	99.1 (0.1)	
Lack of exercise	Yes	85.3 (0.3)	78.5 (0.3)	92.7 (0.2)	<0.001
	No	14.7 (0.3)	21.5 (0.3)	7.3 (0.2)	
Self-perceived socioeconomic status	High	11.1 (0.2)	13.4 (0.3)	8.6 (0.3)	<0.001
	High middle	28.6 (0.3)	28.9 (0.4)	28.1 (0.4)	
	Middle	47.9 (0.3)	45.4 (0.4)	50.5 (0.4)	
	Low middle	10.4 (0.2)	9.8 (0.2)	11.0 (0.3)	
	Low	2.1 (0.1)	2.4 (0.1)	1.8 (0.1)	
Subjective health status	Good–Normal	92.6 (0.1)	94.2 (0.2)	90.8 (0.2)	<0.001
	Poor	7.4 (0.1)	5.8 (0.2)	9.2 (0.2)	
Stress	Yes	80.9 (0.3)	74.9 (0.3)	87.4 (0.3)	<0.001
	No	19.1 (0.3)	25.1 (0.3)	12.6 (0.3)	
Violent events	Yes	2.3 (0.1)	3.2 (0.1)	1.4 (0.1)	<0.001
	No	97.7 (0.1)	96.8 (0.1)	98.6 (0.1)	
Tobacco product use	Never users	86.3 (0.3)	81.0 (0.4)	92.1 (0.3)	<0.001
	Former users	6.5 (0.2)	8.9 (0.2)	3.9 (0.2)	
	Only CC users	3.4 (0.1)	4.3 (0.2)	2.3 (0.2)	
	Only EC users	0.3 (0.0)	0.5 (0.1)	0.1 (0.0)	
	Only HTP users	0.1 (0.0)	0.1 (0.0)	0.1 (0.0)	
	CC + EC users	1.0 (0.1)	1.4 (0.1)	0.5 (0.1)	
	CC + HTP users	0.7 (0.0)	1.1 (0.1)	0.2 (0.0)	
	EC + HTP users	0.2 (0.0)	0.3 (0.0)	0.1 (0.0)	
	CC + EC + HTP users	1.6 (0.1)	2.4 (0.1)	0.7 (0.1)	

Note. CC: combustible cigarettes; EC: electronic cigarettes; HTP: heated tobacco products. Data are presented as percentages (SE).

**Table 2:** Proportion of participants having suicidal thoughts, plans, and attempts according to the type of tobacco use.

		Suicidal Thought (+) (*n* = 7422)		*p*	Suicidal Plan (+) (*n* = 2255)		*p*	Suicidal Attempt (+) (*n* = 1674)		*p*
		N	% (SE)		N	% (SE)		N	% (SE)	
Sex	Boys	2688	9.3 (0.2)	<0.001	874	3.0 (0.1)	<0.001	534	1.8 (0.1)	<0.001
	Girls	4734	17.1 (0.3)		1381	4.9 (0.2)		1140	4.0 (0.1)	
Grade	Middle school	4063	14.0 (0.3)	<0.001	1324	4.5 (0.1)	<0.001	1046	3.5 (0.1)	<0.001
	High school	3359	12.1 (0.3)		931	3.4 (0.1)		628	2.3 (0.1)	
Self-reported scholastic performance	Good–middle	4468	11.5 (0.2)	<0.001	1318	3.4 (0.1)	<0.001	919	2.3 (0.1)	<0.001
	Bad	2954	16.2 (0.3)		937	5.0 (0.2)		755	4.1 (0.2)	
Risky alcohol drinking	Yes	976	22.8 (0.7)	<0.001	411	9.5 (0.5)	<0.001	361	8.4 (0.5)	<0.001
	No	6446	12.2 (0.2)		1844	3.4 (0.1)		1313	2.4 (0.1)	
Substance abuse	Yes	254	47.5 (2.4)	<0.001	177	33.8 (2.5)	<0.001	146	27.4 (2.3)	<0.001
	No	7168	12.7 (0.2)		2078	3.6 (0.1)		1528	2.6 (0.1)	
Lack of exercise	Yes	6377	13.1 (0.2)	0.106	1899	3.9 (0.1)	0.144	1391	2.8 (0.1)	0.016
	No	1045	12.4 (0.4)		366	4.2 (0.2)		283	3.3 (0.2)	
Self-perceived socioeconomic status	High	676	10.9 (0.5)	<0.001	251	4.1 (0.3)	<0.001	178	2.9 (0.2)	<0.001
	High middle	1827	11.4 (0.3)		521	3.1 (0.2)		370	2.3 (0.1)	
	Middle	3297	12.1 (0.3)		928	3.4 (0.1)		667	2.4 (0.1)	
	Low middle	1259	20.7 (0.6)		400	6.5 (0.4)		333	5.3 (0.3)	
	Low	363	29.5 (1.5)		155	12.9 (1.2)		126	9.4 (0.9)	
Subjective health status	Good–Normal	6051	11.4 (0.2)	<0.001	1757	3.3 (0.1)	<0.001	1319	2.4 (0.1)	<0.001
	Poor	1371	33.0 (0.9)		498	11.6 (0.5)		355	8.4 (0.5)	
Stress	Yes	7194	15.6 (0.2)	<0.001	2134	4.6 (0.1)	<0.001	1610	3.4 (0.1)	<0.001
	No	228	2.1 (0.2)		121	1.1 (0.1)		64	0.5 (0.1)	
Violent events	Yes	467	35.7 (1.5)	<0.001	274	21.4 (1.3)	<0.001	226	17.5 (1.3)	<0.001
	No	6955	12.5 (0.2)		1981	3.5 (0.1)		1448	2.5 (0.1)	
Tobacco product use	Never users	5830	11.8 (0.2)	<0.001	1644	3.3 (0.1)	<0.001	1151	2.2 (0.1)	<0.001
	Former users	661	18.3 (0.8)		228	6.2 (0.5)		166	4.4 (0.4)	
	Only CC users	439	22.3 (1.1)		164	7.8 (0.7)		147	7.4 (0.7)	
	Only EC users	33	18.5 (3.1)		17	9.5 (2.5)		14	7.3 (2.0)	
	Only HTP users	17	24.8 (5.9)		8	13.7 (4.6)		9	14.4 (5.2)	
	CC + EC users	120	22.0 (2.2)		38	7.4 (1.3)		34	6.4 (1.2)	
	CC + HTP users	53	14.1 (2.1)		20	4.5 (1.1)		20	4.4 (1.1)	
	EC + HTP users	31	32.3 (5.2)		20	20.1 (4.3)		15	17.6 (4.1)	
	CC + EC + HTP users	238	28.3 (1.7)		116	13.9 (1.3)		118	13.8 (1.4)	

Note. CC: combustible cigarettes; EC: electronic cigarettes; HTP: heated tobacco products; SE: standard error. Data are presented as percentages (standard error). *p*-values were obtained using chi-squared test.

**Table 3:** Association of the type of cigarette with suicidal behaviors.

		OR	95% CI	*p*	OR	95% CI	*p*
Suicidal thoughts (+)	Never users	1					
	Former users	1.82	1.63−2.03	<0.001			
	Only CC users	2.04	1.76−2.37	<0.001	1		
	Only EC users	2.29	1.44−3.65	<0.001	0.98	0.61−1.59	0.940
	Only HTP users	2.47	1.31−4.63	0.005	1.13	0.58−2.23	0.720
	CC + EC users	2.02	1.56−2.62	<0.001	1.01	0.76−1.33	0.959
	CC + HTP users	1.31	0.91−1.90	0.150	0.75	0.51−1.12	0.155
	EC + HTP users	3.91	2.12−7.24	<0.001	1.68	0.90−3.13	0.102
	CC + EC + HTP users	3.08	2.53−3.75	<0.001	1.73	1.41−2.12	<0.001
Suicidal plans (+)	Never users	1					
	Former users	1.89	1.58−2.26	<0.001			
	Only CC users	2.00	1.60−2.51	<0.001	1		
	Only EC users	3.38	1.81−6.33	<0.001	1.55	0.82−2.93	0.178
	Only HTP users	4.13	1.82−9.36	0.001	1.95	0.84−4.52	0.118
	CC + EC users	1.79	1.20−2.66	0.004	0.92	0.60−1.41	0.696
	CC + HTP users	1.26	0.74−2.14	0.398	0.74	0.43−1.28	0.281
	EC + HTP users	6.21	3.14−12.31	<0.001	2.89	1.47−5.66	0.002
	CC + EC + HTP users	3.75	2.86−4.92	<0.001	2.23	1.66−3.00	<0.001
Suicidal attempts (+)	Never users	1					
	Former users	2.10	1.71−2.57	<0.001			
	Only CC users	2.91	2.24−3.76	<0.001	1		
	Only EC users	4.26	2.16−8.38	<0.001	1.49	0.75−2.95	0.259
	Only HTP users	7.13	3.17−16.06	<0.001	2.38	0.98−5.74	0.054
	CC + EC users	2.33	1.55−3.52	<0.001	0.85	0.55−1.31	0.457
	CC + HTP users	2.16	1.25−3.73	0.006	0.85	0.48−1.49	0.569
	EC + HTP users	8.76	4.11−18.70	<0.001	3.00	1.45−6.23	0.003
	CC + EC + HTP users	6.24	4.54−8.56	<0.001	2.59	1.84−3.65	<0.001

Note. CC: combustible cigarettes; EC: electronic cigarettes; HTP: heated tobacco products; OR, odds ratio; CI: confidence interval. Values were calculated by multivariable logistic regression analysis, which was adjusted for sex, grade, subjective health status, self-perceived socioeconomic status, risky alcohol drinking, lack of exercise, scholastic performance, and stress.

## Data Availability

The data that support the findings of this study are openly available in Korea Centers for Disease Control and Prevention at https://www.cdc.go.kr/yhs/home.jsp.
